# Computed tomographic angiography criteria in the diagnosis of brain death—comparison of sensitivity and interobserver reliability of different evaluation scales

**DOI:** 10.1007/s00234-014-1364-9

**Published:** 2014-05-07

**Authors:** Marcin Sawicki, R. Bohatyrewicz, K. Safranow, A. Walecka, J. Walecki, O. Rowinski, J. Solek-Pastuszka, Z. Czajkowski, M. Guzinski, M. Burzynska, J. Wojczal

**Affiliations:** 1 Department of Diagnostic Imaging and Interventional Radiology, Pomeranian Medical University, Clinical Hospital No1, Unii Lubelskiej 1, Szczecin, 71252 Poland; 2 Clinic of Anesthesiology and Intensive Care, Pomeranian Medical University, Szczecin, Poland; 3 Department of Biochemistry and Medical Chemistry, Pomeranian Medical University, Szczecin, Poland; 4 The Centre of Postgraduate Medical Education, Warsaw, Poland; 5 2nd Department of Clinical Radiology, Medical University of Warsaw, Warsaw, Poland; 6 Regional Joint Hospital, Szczecin, Poland; 7 Department of General Radiology, Interventional Radiology and Neuroradiology, Wroclaw Medical University, Wroclaw, Poland; 8 Department of Anesthesiology and Intensive Therapy, Wroclaw Medical University, Wroclaw, Poland; 9 Department of Neurology, Medical University of Lublin, Lublin, Poland

**Keywords:** Brain death, Ancillary test, Computed tomographic angiography, Catheter angiography, Interobserver reliability

## Abstract

**Introduction:**

The standardized diagnostic criteria for computed tomographic angiography (CTA) in diagnosis of brain death (BD) are not yet established. The aim of the study was to compare the sensitivity and interobserver agreement of the three previously used scales of CTA for the diagnosis of BD.

**Methods:**

Eighty-two clinically brain-dead patients underwent CTA with a delay of 40 s after contrast injection. Catheter angiography was used as the reference standard. CTA results were assessed by two radiologists, and the diagnosis of BD was established according to 10-, 7-, and 4-point scales.

**Results:**

Catheter angiography confirmed the diagnosis of BD in all cases. Opacification of certain cerebral vessels as indicator of BD was highly sensitive: cortical segments of the middle cerebral artery (96.3 %), the internal cerebral vein (98.8 %), and the great cerebral vein (98.8 %). Other vessels were less sensitive: the pericallosal artery (74.4 %), cortical segments of the posterior cerebral artery (79.3 %), and the basilar artery (82.9 %). The sensitivities of the 10-, 7-, and 4-point scales were 67.1, 74.4, and 96.3 %, respectively (*p* < 0.001). Percentage interobserver agreement in diagnosis of BD reached 93 % for the 10-point scale, 89 % for the 7-point scale, and 95 % for the 4-point scale (*p* = 0.37).

**Conclusions:**

In the application of CTA to the diagnosis of BD, reducing the assessment of vascular opacification scale from a 10- to a 4-point scale significantly increases the sensitivity and maintains high interobserver reliability.

## Introduction

The use of instrumental tests to confirm brain death (BD) is still the subject of debate [1–3]. In North America, confirmatory testing is not mandatory while in certain European, Central and South American, and Asian countries it is an obligatory part of the procedure to establish BD. Nevertheless, ancillary tests are useful when specific components of the clinical examination cannot be reliably performed. Currently, the most commonly used tests in Europe include electroencephalography (EEG), evoked potentials, transcranial Doppler (TCD), perfusion scintigraphy using ^99m^Tc-HMPAO or ^99m^Tc-ECD, and catheter cerebral angiography. However, all of them have drawbacks related to their feasibility and availability. Electrophysiological tests are susceptible to confounding factors. EEG is unreliable in barbiturate overdose or deep anesthesia and vulnerable to the hostile electrical environment of the ICU. Evoked potentials generally can only be applied in cases of primary supratentorial or secondary brain injuries, because in isolated infratentorial processes they can produce false positive results. Blood flow studies such as TCD, perfusion scintigraphy, and catheter angiography are more feasible but less available in many countries. Therefore, new imaging methods such as magnetic resonance techniques (angiography, spectroscopy, and diffusion-weighted imaging) and positron emission tomography have been proposed [4–7].

Recently, computed tomographic angiography (CTA) attracted attention as a new method for the diagnosis of BD. It appears to be a promising diagnostic alternative that is feasible, widely available, non-invasive, and expeditious. In 1998, Dupas et al. reported the first application of CTA to the diagnosis of BD [8]. Since then, several major studies of this application were published, and national guidelines were introduced in several countries (e.g., in France, Austria, Switzerland, the Netherlands, and Canada) [9–12]. Nevertheless, CTA is not widely accepted as an ancillary test for the diagnosis of BD. The main obstacle is insufficient diagnostic confidence of this method [13]. Until recently, there were no standardized criteria for interpretation of CTA results. The previous major studies assessing CTA in the diagnosis of BD showed that stasis of contrast in the proximal segments of cerebral arteries is frequently found in brain-dead patients, and this phenomenon does not preclude the diagnosis of BD. Based on those observations, three evaluation systems have been proposed: 10-, 7-, and 4-point scales. They include different numbers of intracranial vessels of anterior and posterior cerebral circulation like the 10-point scale or solely anterior circulation like the 7- and 4-point scales. The common criterion of BD in each scale is a lack of opacification of cortical branches of the middle cerebral arteries (MCA-M4) and the deep cerebral veins.

Another issue related to the application of CTA refers to potential difficulty in the assessment of intracranial filling in patients with suspected BD. As previous studies using CTA showed intracranial vessels in these patients are markedly thinner, and their opacification, regardless it’s level, is significantly weaker than normal [14]. Moreover, cerebral vessels are frequently obscured by subarachnoid hemorrhage (SAH) or increased density of subarachnoid space due to pseudosubarachnoid hemorrhage (pseudoSAH) phenomenon, which are frequently found in this group of patients. This can cause difficulties in determining the level of intracranial opacification and produce discrepant diagnoses of BD among radiologists. Therefore, it is reasonable to question interobserver reliability of CTA, especially with the 4-point scale, in which the diagnosis of BD is based on the analysis of only four vessels. Reliability was not addressed in the previous studies, even though it can be useful in the implementation of CTA for the diagnosis of BD.

The aim of the present study was to compare the sensitivity and reliability of the three evaluation scales in CTA for the diagnosis of BD. For this purpose, a single-phase CTA with a delay of 40 s was used. Sensitivity of CTA was compared to the gold standard method—catheter angiography.

## Material and methods

### Study population

In the prospective, multicenter study, 82 patients were examined.

The inclusion criteria were as follows:

1. Deep unresponsive coma (GCS 3–5) of an established etiology capable of causing neurological death.
2. Absence of brainstem reflexes:
  - Pupils mid-position or greater and absent pupillary light reflex (fixed dilated pupils),
  - Spontaneous eye movements,
  - Facial muscle movement to a noxious stimulus,
  - Corneal,
  - Gag/pharyngeal,
  - Cough/tracheal,
  - Oculo-cephalic
  - Vestibulo-ocular (‘cold caloric’).

The exclusion criteria were unresuscitated shock (mean arterial blood pressure ≤ 80 mmHg) and hypothermia (defined as surface temperature ≤ 35 °C). These are known depressants of cerebral blood flow.

The study population consisted of 45 men and 37 women with a mean age of 52.2 years (range, 22–78 years). Initial causes of coma were traumatic brain injury (*n* = 16), intracerebral hemorrhage (*n* = 32), subarachnoid hemorrhage (*n* = 21), ischemic stroke (*n* = 7), and anoxia (*n* = 6). Decompressive craniectomy was performed in 26 (32 %) patients prior to examinations. All patients were managed in the ICUs of two Polish university hospitals and one multi-profile provincial hospital. All subjects underwent cerebral CTA followed by catheter angiography. During all examinations, patients were normoventilated, and mean arterial blood pressure was maintained above 80 mmHg. The elapsed time between the finding of complete brainstem areflexia and the beginning of the radiological examination ranged from 6 through 48 h (not always recorded). Longer delays were usually caused by a prolonged initial observation period due to prior use of sedatives. Shortly, after the radiological studies, the complete clinical testing for determination of BD was performed. According to the Polish national legal regulations, BD was declared when coma, brainstem areflexia, and absence of a breathing drive with 10-min CO_2_ challenge (apnea test) were confirmed. The apnea test result was positive (i.e., supported the clinical diagnosis of BD) if respiratory movements were absent and arterial PCO_2_ was 60 mmHg (or 20 mmHg increase in arterial PCO_2_ over a baseline normal arterial PCO_2_). The clinical test had to be performed twice. Brain death was declared by a commission of three specialists, including at least one in the field of anesthesiology and intensive care and one in the field of neurology or neurosurgery. The majority of brain-dead patients became organ donors. In some cases, organ donation was not possible for medical reasons and occasionally because of a failure to obtain permission from relatives. In such situations, families were informed about the termination of futile therapy, and ventilators were legally discontinued.

### CTA

Two scanners were used to perform CTA: Sensation 64 (Siemens, Erlangen, Germany) and Discovery CT 750 HD (GE Healthcare, Millwaukee, USA). CTA was preceded by a non-enhanced CT scan, which served as a reference. After injecting the contrast, scans were performed with a fixed delay of 40 s. A bolus tracking technique was not used to ensure that this CTA protocol was widely applicable nationwide; this option is not available in all Polish CT facilities. The detailed protocol is presented in Table 1.

**Table 1:** Technical parameters of the CTA methods used for diagnosis of BD

Parameter	Siemens Somatom Sensation 64		GE Discovery CT 750 HD	
	NECT	CTA	NECT	CTA
Acquisition				
Scan range	C5/C6 to vertex			
Tube voltage (KVp)	120	100	120	120
Tube current (mAs)	380	160	320	160
Rotation time (s)	1.0	0.5	1.0	0.6
Collimation (mm)	20 × 0.6	64 × 0.6	32 × 0.625	32 × 0.625
Feed (mm/rotation)	4.8	23.0	16	26.25
Pitch factor	0.8	1.2	0.8	1.2
Scan time (s)	35	6	10	6
Reconstruction				
Slice width (mm)	5.0	0.6	2.5	0.6
Increment (mm)	5.0	0.4	2.5	0.6
Field of view (mm)	250	250	250	250
Matrix	512 × 512	512 × 512	512 × 512	512 × 512
RECON algorithm	H31s	H10f	Standard	Standard
Contrast injection				
Start delay (s)		40		40
Volume (ml)		80		80
Flow rate (ml/s)		4		4
Injection duration (s)		20		20

In the proposed protocol, 80 ml of Iomeron 400 (Bracco Imaging, Konstanz, Germany) was injected at a flow rate of 4 ml/s. This gave injection duration of 20 s. To determine the appropriate delay of scanning, a time to peak concentration of contrast in the head and neck arteries was calculated from the formula proposed by Bae [15]:

$$ {\mathrm{T}}_{\mathrm{peak}}={\mathrm{T}}_{\mathrm{injection}}+8\mathrm{s}=20\mathrm{s}+8\mathrm{s}=28\mathrm{s} $$

T_peak_: Time to peak concentration of contrast in the head and neck arteries
T_injection_: Injection duration.

After adding 15 s to accommodate possible slowed cerebral circulation due to increased cerebrovascular resistance and subtracting a time needed for scanning from the level of C5/C6 to the circle of Willis, the final formula is as follows:

$$ {\mathrm{T}}_{\mathrm{delay}}={\mathrm{T}}_{\mathrm{peak}}+15\mathrm{s}-\frac{{\mathrm{T}}_{\mathrm{scan}}}{2}=28\mathrm{s}+15\mathrm{s}-3\mathrm{s}=40\mathrm{s} $$

T_delay_: Delay of CTA scanning after contrast injection
T_scan_: Scan duration.

All CTA examinations were evaluated by two radiologists, blinded to each other’s assessments, to the results of the clinical test and to the results of the catheter angiography. They had 4 and 8 years of experience interpreting cerebral angiography.

### CTA criteria of BD

Opacification of superficial temporal arteries was assessed to confirm that the contrast was injected correctly and to detect possible hemodynamic perturbations. The following 10 intracranial vessels were evaluated:

- Pericallosal segments of the right and left anterior cerebral artery (ACA-A3),
- Cortical segments of the right and left middle cerebral artery (MCA-M4),
- Cortical segments of the right and left posterior cerebral artery (PCA-P2),
- Basilar artery (BA),
- Right and left internal cerebral vein (ICV),
- Great cerebral vein (GCV)—the vein of Galen.

**Fig. 1:**
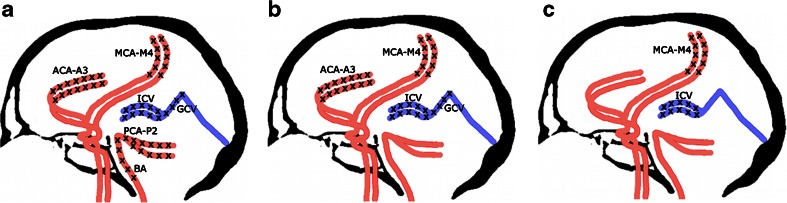
Different criteria for the diagnosis of BD by CTA: **a** Positive result in the 10-point scale (score = 10) confirming the diagnosis of BD was recorded when the following vessels were not opacified: the bilateral PCA-P2, the BA, the bilateral ACA-A3, the bilateral MCA-M4, the bilateral ICV, and the GCV. Scores from 0 to 9 were classified as negative results excluding the diagnosis of BD; **b** In the 7-point scale, positive result (score = 7) was recorded with a lack of opacification of the bilateral ACA-A3, the bilateral MCA-M4, the bilateral ICV, and the GCV. Scores from 0 to 6 were classified as negative results; **c** Positive result in the 4-point scale (score = 4) was recorded when the bilateral MCA-M4 and the bilateral ICV were not opacified. Scores from 0 to 3 were classified as negative results

A point was given for each vessel without opacification, resulting in scores between 0 and 10. Diagnosis of BD was established according to the three previously used evaluation systems: 10-, 7-, and 4-point scales—see Fig. 1. Positive result in each scale confirming the diagnosis of BD was noted with scores 10, 7, and 4, respectively.

### Catheter cerebral angiography

All patients underwent catheter angiography as the second part of the radiological evaluation. Angiography was performed from 15 min to 3 h after CTA. Three angiographic systems were used: Axiom Artis, Fluorospot Top (Siemens, Erlangen, Germany), and MultiDiagnost 3 (Philips, Eindhoven, the Netherlands). The same technique was applied as described previously [16–18]. A typical femoral approach was used in all cases. In 67 patients, aortocervical angiography was used. After positioning a Pigtail 4–5 F catheter in the ascending aorta, 30 ml of contrast was injected at a flow rate of 15 ml/s. In the remaining 15 patients, carotid and vertebral arteries were catheterized selectively; 8 ml of contrast was injected into each artery at a flow rate of 8 ml/s. We visualized the head and neck by recording 50-s series with a frequency of 2 f/s and using a digital subtraction technique. A local neuroradiologist performed and assessed all studies.

### Angiographic criteria of BD

Cerebral circulatory arrest was diagnosed by catheter angiography if either of the following criteria were met:

- Non-filling of intracranial vessels with the normal flow in the external carotid arteries,
- Stasis filling—delayed, weak, and persistent opacification of the proximal cerebral arterial segments without opacification of the cortical branches or venous outflow.

### Data collection

Demographic data recorded for brain-dead patients were age and sex. Clinical data were initial cause of coma, the presence of craniectomy, SAH, or pseudoSAH. PseudoSAH is well-known phenomenon of increased attenuation of the subarachnoid space mimicking SAH. For the diagnosis of BD in CTA and catheter angiography, opacification of the following vessels was recorded:

- Superficial temporal arteries (STA),
- Pericallosal segments of the right and left anterior cerebral artery (ACA-A3),
- Cortical segments of the right and left middle cerebral artery (MCA-M4),
- Cortical segments of the right and left posterior cerebral artery (PCA-P2),
- Basilar artery (BA),
- Right and left internal cerebral vein (ICV),
- Great cerebral vein (GCV)—the vein of Galen.

The presence of stasis filling in CTA and catheter angiography was also noted. Stasis filling was defined as opacification of one or more of the following arterial segments:

- ACA–A1 and A2,
- MCA–M1, M2, and M3,
- PCA–P1 without opacification of the cortical arterial branches or deep veins.

Two radiologists independently evaluated each CTA and recorded the diagnosis of BD using the 10-, 7-, and 4-point scales. A false negative result of CTA was noted when the test precluded BD otherwise confirmed by catheter angiography and clinical testing. A true positive result of CTA was recorded when CTA, catheter angiography, and clinical testing concordantly confirmed BD.

### Statistical analysis

Interobserver agreement was measured by the Kendall’s tau-b rank correlation coefficient for dichotomous variables (diagnosis of BD). The significance of interobserver differences was assessed by McNemar’s test. Cochran’s Q test was used to compare the proportion of false negative results of BD diagnosis between scales. A multivariate logistic regression model with sex, age, presence of craniectomy, and occurrence of SAH/pseudoSAH as independent variables was used to find independent predictors of false negative CTA results. *P* < 0.05 was considered statistically significant. STATISTICA 10 software (StatSoft, Tulsa, USA) was used for the statistical analyses. The tests were evaluated by a medical statistician.

## Results

In all 82 cases, clinical tests confirmed BD. Catheter angiography results were consistent with the diagnosis of BD in all cases, revealing no intracranial filling in 62 (75.6 %) patients with the normal flow in the external carotid arteries. Stasis filling was observed in 20 (24.4 %) patients. This phenomenon was observed from 4 to 48 s after intraarterial injection. No venous outflow was observed in any case.

The analysis of interobserver agreement did not reveal significant differences between the two radiologists who assessed CTA for the diagnosis of BD; see Table 2.

**Table 2:** Interobserver agreement in the diagnosis of BD

Scale	tau-b	Percentage agreement	*p* value
10-point	0.84	93	0.68 (ns)
7-point	0.71	89	0.50 (ns)
4-point	0.64	95	0.13 (ns)

Note: *p* for systematic difference between radiologists (McNemar’s test); *ns* not significant

There were 19 discordant diagnoses of BD between radiologists analyzing CTA. Both radiologists jointly revised each discrepant finding to reach a consensus, which is presented in the following CTA results.

All CTA examinations revealed opacification of the STA with no discrepancies noted between radiologists (percentage agreement was 100 %).

Analysis of opacification of particular cerebral vessels in CTA revealed that they could be classified into the following two groups:

- Frequently opacified vessels: the ACA-A3 (in 21 patients), PCA-P2 (17), and BA (14),
- Rarely opacified vessels: MCA-M4 (3 patients), ICV (1), and GCV (1), see Table 3.

**Table 3:** Patient’s characteristics and imaging findings in cases of false negative CTA results

#	Sex	Age	Cause of BD	CTA opacification										BD diagnosis			Craniectomy	SAH or pseudoSAH	Stasis filling in CA
				MCA-M4 right	MCA-M4 left	ACA-A3 right	ACA-A3 left	PCA-P2 right	PCA-P2 left	BA	ICV right	ICV left	GCV	10-point	7-point	4-point			
1	M	56	ICH	1	1	0	0	1	1	1	1	1	1	n	n	y	−	−	−
2	M	71	ICS	0	0	0	0	0	0	1	0	1	0	n	n	n	+	+	+
4	M	73	TBI	1	0	0	0	0	0	0	1	1	1	n	n	n	+	+	+
6	F	49	SAH	1	1	1	1	1	1	0	1	1	1	n	y	y	−	+	−
16	F	47	SAH	1	1	0	0	1	1	1	1	1	1	n	n	y	−	+	+
18	M	53	TBI	1	1	0	0	0	0	0	1	1	1	n	n	y	+	+	−
22	F	67	ICH	1	1	0	0	1	1	1	1	1	1	n	n	y	−	+	+
25	M	63	ANX	1	1	1	1	1	1	0	1	1	1	n	y	y	+	+	−
35	M	78	ICH	1	1	0	0	1	1	0	1	1	1	n	n	y	+	+	+
36	M	39	ICH	1	1	1	0	1	1	1	1	1	1	n	n	y	+	+	+
39	F	74	TBI	1	1	0	0	1	1	1	1	1	1	n	n	y	−	+	+
41	F	40	SAH	1	1	0	0	0	0	1	1	1	1	n	n	y	+	+	+
42	F	22	ICS	1	1	0	0	0	1	1	1	1	1	n	n	y	+	+	+
43	F	44	ICH	1	1	0	0	1	1	1	1	1	1	n	n	y	+	+	+
44	F	34	ICH	0	0	0	0	0	0	1	1	1	1	n	n	n	+	+	+
45	M	50	TBI	1	1	1	1	0	0	0	1	1	1	n	y	y	+	+	−
46	M	44	SAH	1	1	0	0	0	1	1	1	1	1	n	n	y	+	+	+
47	M	51	ICH	1	1	0	0	0	0	0	1	1	1	n	n	y	+	−	+
50	M	71	ANX	1	1	0	0	0	0	1	1	1	1	n	n	y	+	+	−
55	M	59	TBI	1	1	0	0	0	0	0	1	1	1	n	n	y	+	−	−
59	M	62	SAH	1	1	0	0	0	0	1	1	1	1	n	n	y	+	+	−
60	M	56	ICH	1	1	0	0	0	0	0	1	1	1	n	n	y	−	+	+
62	M	65	SAH	1	1	1	1	0	0	0	1	1	1	n	y	y	+	+	−
67	M	63	ICH	1	1	1	1	1	1	0	1	1	1	n	y	y	−	+	−
77	M	54	ICH	1	1	0	0	0	0	0	1	1	1	n	n	y	−	−	+
80	F	49	ICH	1	1	0	0	0	0	0	1	1	1	n	n	y	−	+	+
82	M	61	ICH	1	1	1	1	0	0	0	1	1	1	n	y	y	−	+	−

Note: Cause of BD: *ICH* intracerebral hemorrhage; *ICS* ischemic stroke; *TBI* traumatic brain injury; *SAH* subarachnoid hemorrhage; *ANX* anoxia

CTA opacification: 1–no opacification; 0–vessel opacified

BD diagnosis: y–CTA criteria fulfilled; *n*–criteria not fulfilled

*CA* catheter angiography

**Fig. 2:**
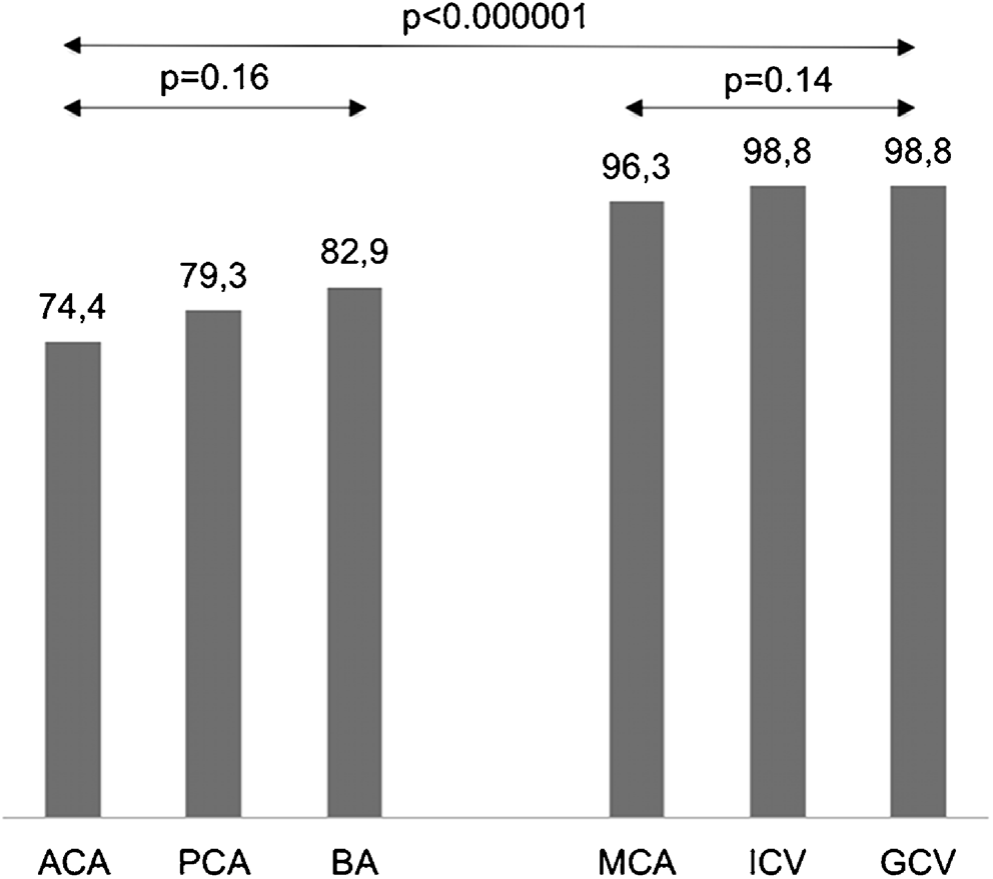
Sensitivity of cerebral vessels as indicators of cerebral circulatory arrest in CTA. All vessels were classified into the high sensitivity group (cortical segments of the MCA, ICV, and GCV) and the low sensitivity group (the pericallosal artery and cortical segments of the PCA and BA)

Regarding their sensitivity as indicators of cerebral circulatory arrest, the high sensitivity group included cortical segments of the MCA, ICV, and GCV. The low sensitivity group consisted of the pericallosal artery and cortical segments of the PCA and BA. The sensitivities of all six vessels differed significantly (*p* < 0.001, Cochran’s Q test). There were no significant differences between vessels within the high sensitivity group and the low sensitivity group, see Fig. 2.

**Fig. 3:**
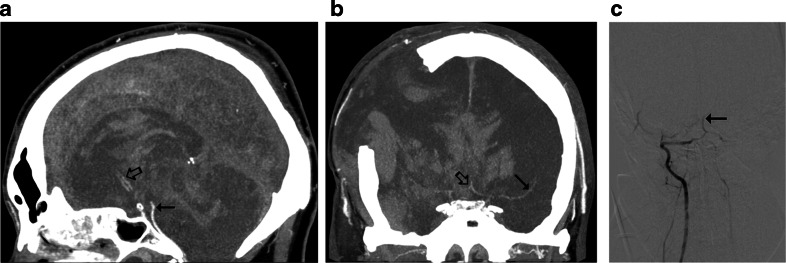
CTA findings in a 50-year-old man (patient no. 45) with traumatic brain injury (epidural hematoma in the right parietal region, massive intracerebral, and subarachnoid and intraventricular hemorrhage) and right sided craniectomy presented with signs of BD on clinical examination: **a** Ten millimeter maximum intensity projection (MIP) in sagittal plane. CTA shows opacification of the BA (*thin arrow*) and a trace of contrast in A2 segments of the ACAs (*thick arrow*). **b** Ten millimeter MIP in coronal plane. CTA shows opacification of the M1 segment of the left MCA (*thin arrow*) and the A1 segments of the ACAs (*thick arrow*); these findings exclude the diagnosis of BD according to the 10-point scale but confirm BD according to the 7- and 4-point scales. **c** Catheter angiography of the right VA performed 0.5 h later revealed delayed, residual filling of the BA (*arrow*) that occurred 21 s after injection. This result was interpreted as stasis filling consistent with the diagnosis of BD

**Fig. 4:**
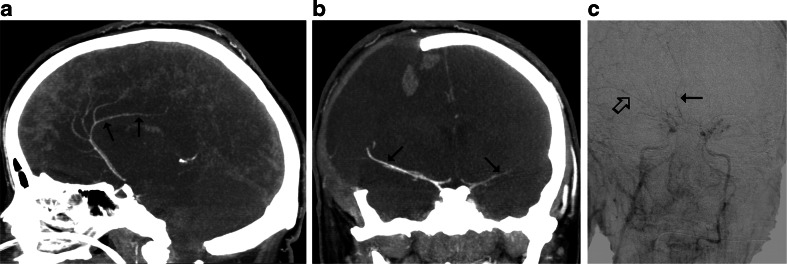
CTA findings in a 22-year-old woman (patient no. 42) with a brain stem ischemic stroke and a right sided craniectomy who presented with signs of BD on clinical examination. **a** Ten millimeter MIP in sagittal plane. CTA shows opacification of the right pericallosal artery (thin arrows); **b** Ten millimeter MIP in coronal plane. CTA shows opacification of the M1 segments of the MCAs (*thin arrows*). These findings exclude the diagnosis of BD according to the 10- and 7-point scales but confirm BD according to the 4-point scale. **c** Catheter angiography from the aortic arch performed 1 h later revealed delayed, residual filling of the M1 segment of the right MCA (*thick arrow*) and A2 segment of the right ACA (*thin arrow*) that occurred 32 s after injection. This result was interpreted as stasis filling consistent with the diagnosis of BD

**Fig. 5:**
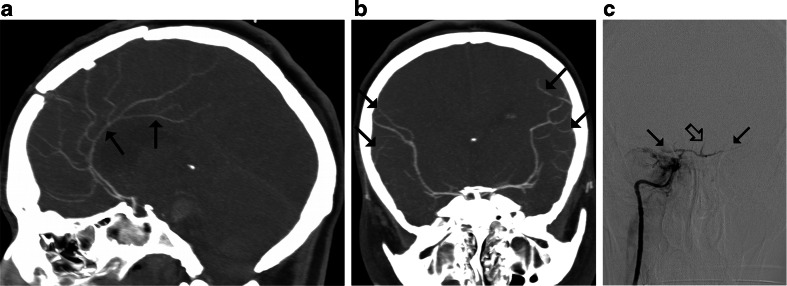
CTA findings in a 34-year-old woman (patient no. 44) with brain stem hematoma and frontal craniotomy presenting signs of BD on clinical examination. **a** Ten millimeter MIP in sagittal plane. CTA shows opacification of both pericallosal arteries (*thin arrows*); **b** Ten millimeter MIP in coronal plane. CTA shows opacification of the cortical segments of the MCAs (*thin arrows*); these findings exclude the diagnosis of BD according to the 10-, 7-, and 4-point scales. **c** Catheter angiography of the right ICA performed 3 h later revealed delayed, residual filling of the M1 segments of the MCAs (*thin arrows*) and the A2 segments of the ACAs (*thick arrow*) that occurred 18 s after injection. This result was interpreted as stasis filling consistent with the diagnosis of BD

**Fig. 6:**
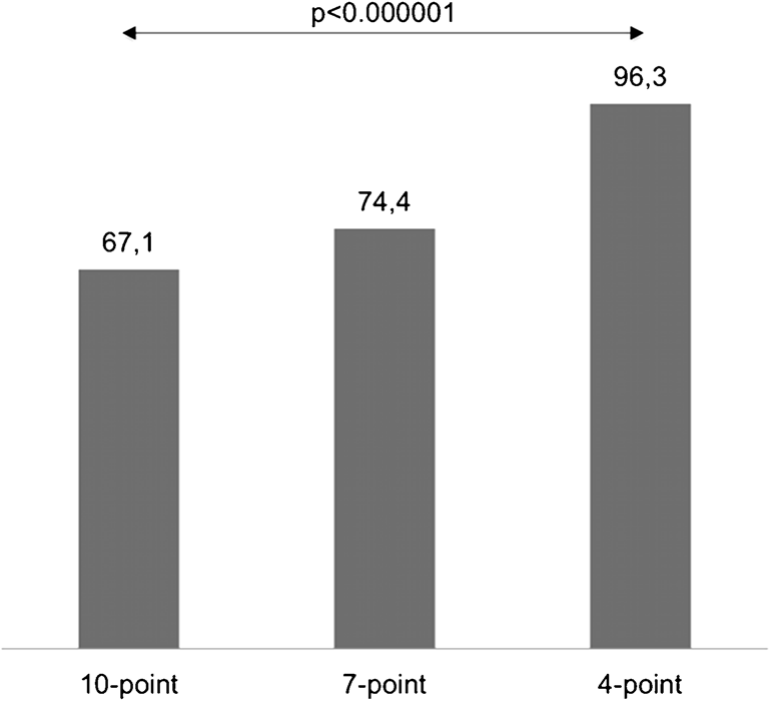
Sensitivity of three CTA scales for the diagnosis of BD. The sensitivities differed significantly (*p* < 0.001, Cochran’s Q test)

According to the 10-point scale, 27 false negative CTA results were observed, see Fig. 3. With the 7-point scale, the number of false negative results decreased to 21, see Fig. 4. The 4-point scale provided three false negative results, see Fig. 5. A comparison of sensitivities for different CTA scales is presented in Fig. 6.

According to the 10-point scale, CTA showed a non-filling pattern in 32 (39 %) patients and stasis filling in 23 (28 %) patients.

Sex, age, presence of craniectomy, and the occurrence of SAH/pseudoSAH were analyzed as independent predictors of a false negative CTA result in the 10-point scale. Presence of craniectomy appeared to be an independent predictive factor of a false negative CTA. Relative risk of a false negative CTA in patients with craniectomy was 3.13 times higher than in those without it (95 % CI = 1.70 to 5.77; *p* = 0.0002). The second independent predictor of a false negative CTA was the presence of SAH/pseudoSAH observed in 54 patients. Relative risk of a false negative CTA in patients with SAH/pseudoSAH was 2.98 times higher than in those without it (95 % CI = 1.14 to 7.78; *p* = 0.0255). Sex and age were not associated with false negative CTA results.

## Discussion

The percents of agreement between the radiologists were equally high (89–95 %) for all scales. The fact that the observers did not reach perfect agreement could be explained by significantly lower contrast enhancement of cerebral arteries in brain-dead patients compared to normal opacification [14]. However, the analysis of interobserver agreement showed a trend towards lower values of Kendall’s tau-b coefficient using the 4-point scale. Interobserver agreement coefficients, including tau-b, are affected by the prevalence of measures under consideration; therefore, low values of tau-b may be due to rare findings, such as negative CTA results using the 4-point scale, may not reflect low rates of agreement. This is a known paradox explained by Feinstein and Cicchetti [19, 20]. In such situations, the percentage agreement is more reliable indicator of interobserver concordance.

Concerning opacification of individual cerebral vessels as indicators of cerebral circulatory arrest, two groups can be distinguished:

- High sensitivity: MCA, ICV, and GCV,
- Low sensitivity: ACA, PCA, and BA.

In previous studies, different sets of vessels were considered in the diagnosis of cerebral circulatory arrest [8, 21–26]. The first evaluation scale was introduced by Dupas et al. in 1998 [8]. They observed 100 % sensitivity and specificity of the pericallosal artery, MCA-M4, ICV, and GCV, and proposed the 7-point system, which excluded arteries of the posterior cerebral circulation, because their sensitivity was lower (85.7 % for BA). Delayed opacification of the BA in clinically brain-dead patients was observed in several studies using catheter angiography [27–31]. Braun et al. reported this phenomenon in 24 (17 %) of 140 patients meeting clinical criteria of BD; these findings were verified by positive TCD in six cases [31]. Only four previous studies assessed opacification of the BA with CTA in BD [8, 21, 23, 25]. Combes et al. reported a 100 % sensitivity of the BA [25]; however, the other authors observed sensitivities that ranged from 86 to 94 %. These later results are consistent with our finding for the BA, 82.9 % sensitivity. Simultaneously, in all cases, catheter angiography showed a lack of opacification of BA in the 30- to 50-s series. In the study by Welschehold et al., all cases were verified by a positive TCD result [21]. These findings show that opacification of BA can be observed in up to 17 % of brain-dead patients and does not indicate preserved cerebral perfusion, thus does not preclude diagnosis of BD. A high frequency of BA opacification in brain-dead patients can be explained by the protective role of the cerebellar tentorium. The majority of primary brain injuries affect the supratentorial compartment; therefore, increasing intracranial pressure (ICP) primarily stops capillary and venous flow to the cerebral hemispheres. In these injuries, ICP may rise more slowly in the infratentorial region, allowing contrast to enter arteries of posterior cerebral circulation. Cessation of flow in posterior cranial fossa can occur with a delay, providing false negative results of blood flow examinations. One of such cases is presented in Fig. 3.

In regard to the diagnostic value of pericallosal artery, the 100 % sensitivity observed by Dupas et al. was not confirmed in other studies that reported a sensitivity of 57–83 % for this artery [8, 21–25]. In this study, the pericallosal artery had the lowest level among all the vessels evaluated. The shorter distance from the skull base to the pericallosal artery, in comparison with the MCA-M4, can explain the lower sensitivity of the ACA-A3 for the CTA diagnosis of BD. According to Poisseuille’s equation, cerebral blood flow (CBF) is inversely proportional to the length of the vessel.

$$ \mathrm{CBF}=\frac{\pi \times \mathrm{CPP}\times {\mathrm{radius}}^4}{8\times \mathrm{viscosity}\times \mathrm{length}} $$

CPP: Cerebral perfusion pressure
Radius: Radius of the vessel
Viscosity: Viscosity of the blood
Length: Length of the vessel.

Therefore, raised ICP primarily stops the flow in the distal cortical segments of the MCA, whereas the contrasts column continues to reach more proximal pericallosal arteries for some time. One such case is presented in Fig. 4.

A high frequency of opacification of ACA-A3 in brain-dead patients is the principal cause of the low sensitivity of CTA in the 10- and 7-point scales previously reported and confirmed by this study, see Table 4.

**Table 4:** Results of the major studies that evaluated the sensitivity of CTA for the diagnosis of BD by the type of scale utilized

Study authors and year	Sensitivity (%)		
	10-point	7-point	4-point
Combes et al. 2007 [25]	70		
Welschehold et al. * 2013 [21]	54		
Dupas et al. 1998 [8]		100	
Quesnel et al. ** 2007 [24]		52	
Frampas et al. 2009 [23]		63	86
Rieke et al. 2011 [22]		76	93
Leclerc et al. 2006 [26]			87
Present study	67.1	74.4	96.3

Note: *GCV was not assessed, **This study included 5 (of 21) patients with anoxic brain injury, which predisposes to preserved residual perfusion

In 2006, Leclerc et al. introduced the 4-point scale, which is limited to the most sensitive parameters, the MCA and ICV, and excludes the ACA and GCV [26]. Exclusion of GCV was motivated by the fact that it drains blood partially from the posterior circulation. Therefore, this was a consequence of exclusion of the BA and PCA. In addition, circulation to the basal ganglia is assessed by opacification of the ICVs; therefore, opacification of the GCV, a confluence of the ICVs, does not provide any additional information, despite its high individual sensitivity. This approach was included in the recommendations of the French Society of Neuroradiology. The GCV was included in their earlier, 2007, recommendations, but was excluded from their most recent, 2011, revised recommendations [12, 32]. To summarize, evaluation of CTA in the diagnosis of BD evolved from the 10- and 7-point systems to the more highly sensitive 4-point scale.

It must be noted that respecting the widely accepted concept of the whole brain death, an ancillary test should assess the perfusion of the entire brain including anterior and posterior circulation. This condition is not fulfilled in the 7- and 4-point scales limited to anterior circulation. However, some established ancillary tests do not entirely address this issue either. TCD for the diagnosis of BD consists of assessing blood flow in MCAs and BA, omitting ACAs and PCAs. There are opinions that in planar radionuclide perfusion examination it is difficult to evaluate the perfusion of the brainstem because of overlying parotid gland and other soft tissues [33]. Evoked potentials allow examination of specific areas of interest in the brainstem. Finally, EEG records activity from only the cortical layers immediately beneath the scalp; it does not record from subcortical structures, such as the brainstem or thalamus. It should be considered that ancillary test, even if limited to assessment of the anterior circulation, is always combined with the clinical examination testing brainstem function. Besides, no false positive result of CTA according to 7- or 4-point scale has been reported so far (i.e., CTA confirmed BD, yet the patient survived).

While analyzing a relationship between demographic and clinical features, and the sensitivity of CTA, it was found that craniectomy independently predisposed to false negative results. Preserved intracranial opacification in brain-dead patients with skull defects, particularly craniectomy, was previously reported [22, 23, 30, 31, 34]. Three of such cases are presented in Figs. 3, 4, and 5. This can be explained by regional reduction of ICP caused by a skull defect. Alvarez et al. postulated that intracranial hypertension is nevertheless severe enough to induce cerebral circulatory arrest in these cases [30]. However, the residual cerebral flow at the region of the skull defect is a major limitation of perfusion tests for confirmation of BD. Frampas et al. recommended a minimum observation period of 6 h before CTA in this clinical situation because of the slow increase of ICP [23].

SAH or pseudoSAH appeared to be a second factor that predisposed to false negative CTA results. This finding may be explained by increased baseline density of the subarachnoid space in patients with SAH or pseudoSAH, which can be mistaken for a true vascular opacification. These findings support the observation of Gutierrez et al. that interpretation of CT can be difficult in such circumstances [35].

In many patients, catheter angiography showed more proximal intracranial opacification than did CTA. Dupas et al. observed the same divergence in four of seven cases [8]. A larger volume of contrast was used in CTA than in catheter angiography, but during angiography, contrast was injected intra-arterially, which minimizes the dilutional effect. This discrepancy could have resulted from the sequence in which CTA and catheter angiography were performed. Angiography was performed from 15 min to 3 h after CTA. During this time interval, a slowly rising ICP could further suppress cerebral circulation and stop contrast movement at more proximal levels.

The proposed CTA protocol differs from the standard technique of CTA in the diagnosis of BD consisting of NECT and two post-contrast phases started at 20 and 60 s after injection [36]. The 20- to 60-s protocol was proposed by Dupas et al. in 1998 [8] and was applied in several later studies [21–26]. However, in clinical practice, particularly in France, it is routinely combined with whole body CT for assessing transplantable organs with a single contrast injection. Such combination requires a larger volume of contrast (120 ml instead of ≈80 ml) and longer injection duration (40 s instead of ≈20 s) in comparison to a standard CTA of the brain.

The technique proposed in the present study is dedicated for assessing cerebral vessels, as whole body CT was not included in the examination. Therefore, a volume of contrast was reduced to 80 ml—a standard for cerebral CTA. A standard flow rate of 4 ml/s was used as well. This gave injection duration of 20 s—twice as shorter in comparison to the 20- to 60-s protocol. While choosing a proper delay of CTA scanning for the diagnosis of BD, one must take into account that in some cases cerebral circulation may be slowed due to intracranial hypertension. A key issue is to cover this delayed vascular opacification with CTA scanning. As perfusion and angiographic studies show, there is a threshold time of approximately 15 s for cerebral circulation to preserve a viability of the brain [28, 37–41]. With longer circulation time, an opacification of cerebral vessels does not indicate preserved brain perfusion thus does not preclude the diagnosis of BD. Thus, optimal CTA scan for the diagnosis of BD should be performed approximately 15 s after peak concentration of contrast is achieved in the head and neck arteries. This time is 40 s after injection with the injection duration of 20 s used in the present study. It should be noted that the standard 60-s phase is also performed approximately 15 s after peak concentration of contrast in the head and neck arteries, because the standard protocol includes the 40-s injection duration.

Applying the injection duration of 20 s and following the standard protocol with scanning at 60 s after injection would be consistent with assessing vascular opacification over 30 s after peak contrast concentration in the head and neck arteries. This would detect extremely slow cerebral circulation not indicative of preserved perfusion but providing false negative results and decreasing the sensitivity of CTA.

Another difference between the proposed protocol and the 20- to 60-s technique is an abandonment of the early 20-s phase. The early phase is used for assessing of opacification of extracranial arteries as an indicator of sufficient contrast delivery. However, the study by Leclerc et al. showed that extracranial arteries are similarly opacified in the 20-s and in the 60-s phase [26]. They postulated that the scanning protocol could be limited to a single, 60-s phase. Therefore, it was reasonable to assume that scanning with a delay of 40 s will be sufficient to detect opacification of extracranial arteries. The results confirmed these assumptions because all CTA examinations revealed sufficient opacification of the STA. The proposed single-phase protocol can be a feasible alternative to the two-phase technique in the diagnosis of BD, when whole body CT is not simultaneously performed. In comparison to the two-phase protocol, the single-phase method is simpler to perform and requires a lower volume of contrast, which is less harmful for the kidneys. However, the accuracy of both protocols needs to be comparatively evaluated.

## Limitations

The study was limited to one measure, sensitivity, of the accuracy of CTA for the diagnosis of BD. To more completely describe the accuracy of CTA for the diagnosis of BD, assessment of its specificity is needed, preferably with a control group, including comatose patients at different degrees of intracranial hypertension.

## Conclusions

In the application of CTA for the diagnosis of BD, reducing the assessment of vascular opacification scale from a 10- to a 4-point scale significantly increases the sensitivity and maintains high interobserver reliability. Therefore, the 4-point scale seems to be optimal in the determination of BD. Nevertheless, introduction of CTA as reliable ancillary test cannot be recommended without evaluation of its specificity, which has not been comprehensively studied yet.
